# MSF: Modulated Sub-graph Finder

**DOI:** 10.12688/f1000research.16005.3

**Published:** 2019-04-14

**Authors:** Mariam R. Farman, Ivo L. Hofacker, Fabian Amman

**Affiliations:** 1 Institute for Theoretical Chemistry,Theoretical Biochemistry Group,, University of Vienna, Vienna, 1090, Austria; 2 Department of Chromosome Biology, Max F. Perutz Laboratories,, University of Vienna, Vienna, 1030, Austria

**Keywords:** Differential gene expression analysis, pathway analysis, combining p-value, cell signalling network

## Abstract

High throughput techniques such as RNA-seq or microarray analysis have proven to be invaluable for the characterizing of global transcriptional gene activity changes due to external stimuli or diseases. Differential gene expression analysis (DGEA) is the first step in the course of data interpretation, typically producing lists of dozens to thousands of differentially expressed genes. To further guide the interpretation of these lists, different pathway analysis approaches have been developed. These tools typically rely on the classification of genes into sets of genes, such as pathways, based on the interactions between the genes and their function in a common biological process. Regardless of technical differences, these methods do not properly account for cross talk between different pathways and most of the methods rely on binary separation into differentially expressed gene and unaffected genes based on an arbitrarily set *p*-value cut-off.

To overcome this limitation, we developed a novel approach to identify concertedly modulated sub-graphs in the global cell signaling network, based on the DGEA results of all genes tested. To this end, expression patterns of genes are integrated according to the topology of their interactions and allow potentially to read the flow of information and identify the effectors. The described software, named Modulated Sub-graph Finder (MSF) is freely available at https://github.com/Modulated-Subgraph-Finder/MSF.

## Introduction

High throughput sequencing techniques have been widely used to yield differentially expressed genes (DEG) ^1^. The changes in transcript abundance are measured, e.g. by next generation sequencing techniques and interpreted as an indicator of differential expression of genes. DEGs can be used to gain insights into the mechanisms underlying differences between conditions of samples, such as healthy versus infected. Differential gene expression analysis (DGEA) informs about the magnitude of expression changes, which are often expressed as log-fold change. The sign of log-fold change and the confidence level of observing an authentic change, often expressed as *p*-value. The information from DEGs is further interpreted to extract meaningful biological insights. For example, genes that could be involved in the response to a particular stimulus. To this end, pathway-based analysis has become an important tool to further interpret the results of a DGEA and to acquire understandings of the perturbations in a biological system. These pathway-based methods use predefined pathways or networks which are sets of genes with their interactions forming a functional unit. DEGs help to identify pathways or networks that may be altered during an infection, providing important information about diseases and its treatment process ^2^. The expression measurements of the genes obtained from DGEA in combination with statistical methods and the predefined pathways are used to identify specifically modulated pathways and processes ^3^.

Well established resources for pathway annotations are KEGG (Kyoto Encyclopedia of Genes and Genomes) ^4^ and Reactome ^5^. KEGG pathways is a branch of KEGG database that hosts a collection of manually drawn pathway maps representing the molecular interaction, reaction and relation networks of cellular functions. Similarly, Reactome is an open-source, manually curated, peer-reviewed database for signaling and metabolic molecules with their interactions formed into different biological pathways ^5^. Both provide predefined pathways which are sets of genes and their interactions categorized into functional units.

Existing pathway-based analysis approaches use different research designs, which can be categorized into ORA (Over-representation analysis), FCS (Functional class scoring) and pathway topology based methods. All of which aim to find a subset of genes, e.g., significantly differentially expressed genes, genes associated with a certain pathway more often than expected given the total set of examined genes, e.g. the whole genome background. ORA is the first and the most basic method of pathway analysis ^3^. It uses a DEG list with user defined cut-off for the log-fold change and *p*-value (most commonly using absolute log-fold change *≥* 2 and *p*-value *≤* 0.05). Subsequently, sets of genes associated with annotated pathways are tested for being over-represented in the set of DEGs. To this end, hyper-geometric distribution, chi-square tests, binomial probability or the Fisher’s exact test are used, whereas, the information of the topology of genes in the pathways is neglected ^6^. Furthermore, ORA assumes that the biological pathways are independent of each other and ignores the fact that they cross-talk and overlap ^2, 3^.

Unlike ORA, FCS has no artificial cut-off to define a DEG list. FCS works in three steps. First it calculates the gene-level statistics including correlation of molecular measurements using differential expression of individual genes, ANOVA, t-test and Z-test. In the second step the statistics of individual genes in a pathway are transformed to an individual pathway-level statistic commonly using Kolmogorov-Smirnov statistic, mean or median. Finally the statistical significance of the pathway-level statistics is assessed. Although FCS overcomes some of the limitations from ORA, it still ignores the topology of genes in a pathway, cross-talk and overlap of the pathways ^2, 3^. Pathway topology based methods are similar to FCS except that they consider the topology of each gene during the gene-level statistics but still lacks to link the different functional pathways ^2^.

From another perspective, network based approaches do not categorize sets of genes into functional pathways, but they consider all interactions to be equal. Thereby, they avoid distinguishing arbitrarily between interactions within a pathway and interactions between pathways (i.e., cross-talk). With this they aim to identify subnetworks that show modulation between two conditions or upon a stimuli ^7^. To find these active modules heuristics solutions like simulated annealing (SA), greedy methods, genetic algorithms (GA), network propagation and co-clustering methods are used ^7^. jActiveModules has been the first of this kind using simulated annealing to find modulated sub-networks ^8^. The benefit of omitting pathways is brought by reduced interpretability of the results due to the lack of functional labels on the networks.

On these grounds we propose a novel approach to make use of the rich gene and protein interaction annotation resources available and combining it to functional pathway annotations to gain additional insights from basic DGEA. To this, we start with the presupposition that expression of neighboring genes within a functional pathway are not independent from each other. Rather, they are often regulating each other’s expression or are part of the same regulon ^9^. We understand that the categorization of links between genes into labeled pathways is often an arbitrary one, given the extensive cross talk between different pathways. Although these categories have proven to be useful in many situations, they force a certain perspective onto the interpretation of novel data. Based on these two principles, we aim to find sub-graphs of connected genes within cell signaling network, which exhibit as a whole significant differential expression changes. This approach differs in two main aspects from common pathway analysis tools. First, it does not aim to identify functional pathways enriched in differentially expressed genes, but detects sub-graphs or branches in a network graph (potentially spanning more than one functionally grouped pathway) which is coherently modulated. Second, it aims to improve the DGEA on the gene level, by collecting the information of neighboring genes, which as a whole might exhibit prominent enough signal to be called significantly modulated. All of this can be helpful to understand the cause and effect of a stimulus and might inform about potential points of intervention.

As input, information on functional links between genes provided by e.g. KEGG or Reactome and information on the differential expression status of single genes resulting from a DGEA, are required. As a result the analysis returns sub-graphs and their joint confidence scores, reflecting how the perturbation migrates through the network. Furthermore, the entry points of perturbation in the networks and overlap with conventional pathway categories are returned. To facilitate prioritization of the perturbation entry points, to each an impact score and a measure of its reliability are assigned. The impact score expresses the fraction of the sub-module being downstream of the entry point. The reliability is measured using a t-test on log transformed *p*-values of the immediate upstream and downstream genes. The output is prepared in a directed adjacency matrix file, convenient for visualization, e.g., with StringApp ^10^, available as a Cytoscape plug-in ^11^.

The proposed algorithm is named Modulated Sub-graph Finder (abbreviated MSF). MSF can help transform the information obtained from DGEA into comprehensible knowledge of signal transduction of genes, hence being a valuable complement to existing pathway based methods. MSF is freely accessible on GitHub under the terms of the Creative Commons Attribution 4.0 International License.

## Methods

MSF was implemented as a Java program. It is developed as a novel heuristic approach to find concertedly modulated sub-graphs in networks of biological interactions. MSF does not use predefined gene sets grouped into functional units, but rather relies purely on the network of interacting genes. The input network consists of nodes corresponding to genes and edges representing interactions. Furthermore it utilizes comprehensive results from a differential gene expression analysis to discover the sub-graphs, or modules, which are as a whole modulated.

MSF uses the individual gene’s *p*-values generated from the DGEA. The *p*-value expresses the probability that the null hypothesis of unmodified gene expression can’t be rejected for a given statistical model. To find significantly modulated sub-graphs individual *p*-values of the vicinal genes in the global network are combined into a single combined *p*-value, using a statistical method for combining dependent *p*-values described by Hartung ^12^. Hartung’s method uses the inverse of standard normal distribution function. Using the inverse normal cumulative distribution function Φ ^−1^, individual gene *p*-values *T _i_* are transformed to their corresponding normal score *t _i_* = Φ ^−1^(1 − *T _i_* ) that is uniformly distributed on (0,1). Then using these normal scores, the correlation between genes is calculated *Cov*( *t _i_*, *t _j_* ) = *ρ*. The normal scores and correlation are applied to the weighted inverse normal function to calculate the combined *p*-value *t*( *ρ*) for all genes examined, namely the examined sub-graph

$$t(\rho )=\frac{\sum_{i=1}^{n}\lambda_{i}t_{i}}{\sqrt{(1-\rho )\sum_{i=1}^{n}\lambda_{i}^{2}+\rho {\left({\sum_{i=1}^{n}\lambda }_{i}\right)}^{2}}}$$

Lambda *λ* be the weights for each gene, currently all genes have equal weight, i.e. 1. The combined *p*-value *t*( *ρ*) of a sub-graph will express the significance of all genes in the sub-graph being modulated together. The information from the different genes are used as, although not independent, replicated measurement of the behavior of the whole sub-graph. This potentially increases the power to detect also significant sub-graphs consisting of genes which are not significant on there own.

### Overview of our method

**Figure 1.**
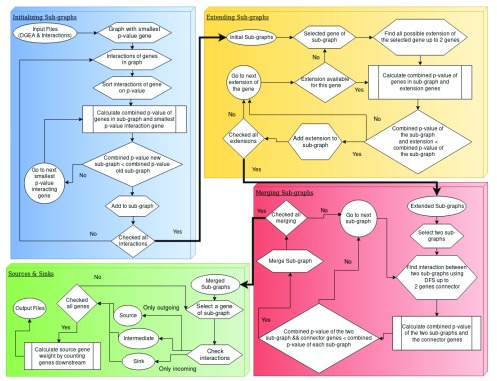
Graphical representation of the MSF heuristic approach to detect modulated sub-graphs in a global gene regulatory network.

To reduce the complexity to score all possible connected sub-graphs MSF applies a four step heuristic as described in the following. The proceeding identification of modulated sub-graphs from a network by MSF is presented as a flowchart diagram ( Figure 1).

***Initializing modulated sub-graphs.*** MSF constructs the first sub-graph starting with the genes associated with the lowest (most significant) *p*-value deduced from the DGEA. From this seed it tries to extend the sub-graph by adding directly neighboring genes, starting with the next most significant one. A single combined *p*-value is calculated for the extended sub-graph. If the combined *p*-value is smaller than the *p*-value of the original sub-graph, the extended sub-graph is accepted. This step is iteratively repeated until no further extension is accepted. In this case the process starts over with all remaining genes not yet in the significantly modulated sub-graph. This step identifies all simple sub-graphs that are modulated in the whole network.

***Extending modulated sub-graphs.*** In the next step, we check if any of the initial modulated sub-graphs could further be extended beyond the immediate neighboring genes. Instead of testing single genes and their compatibility to be added, groups of genes are considered. If the combined *p*-value of the initial modulated sub-graph and the extension genes is smaller than the *p*-value of the initial sub-graph the extension is accepted. All possible extension paths up to *3* (default 2) genes at all nodes in the sub-graph are tested. Again, this step is iteratively repeated until no further genes are added to the significant differentially expressed sub-graphs. This step bridges small gaps of genes without a clear differential signal in the DGEA.

***Merging modulated sub-graphs.*** After detection and extension of the modulated sub-graphs, each pair of so far identified sub-graphs is tested if its combination scores better than each on its own. The merging of the sub-graphs is done by depth first search traversal from one sub-graph to the other sub-graph. If the two sub-graphs merge with the connector of at most *3* genes (default 2 genes) and the combined *p*-value of the merged sub-graph including the bridging genes in between is less than the individual *p*-values of the two sub-graphs, the two sub-graphs are merged together to one bigger modulated sub-graph. This step is repeated iteratively until no sub-graphs can be merged anymore.

***Finding sources & sinks.*** In a last post processing step MSF identifies the trigger points of the modulated sub-graphs. These trigger genes are the sources of the sub-graphs with only outgoing edges. These genes can be interpreted as the possible entry points of perturbation from where the stimulus causes downstream effects. Each individual source is given an impact score, expressing the relative number of downstream genes within the corresponding sub-graph directly connected by directed links. This score can be interpreted as an upper limit of how much of the sub-graphs’ perturbation could have been introduced by the respective source, and thereby could be helpful to prioritize different identified sources for larger sub-graphs. In the same spirit as sources, the most downstream genes of the modulated sub-graphs are identified and defined as sinks. Due to loops not all sub-graphs are guaranteed to have sources or sinks. The reliability of each source is inspected using a t-test on log transformed *p*-values. The significant difference between the two groups, genes downstream the source and the genes upstream of the source is determined. This would help to assess if the source identified is reliable and indeed marks the border between two different regulation regimes.

***MSF output.*** MSF generates a directed network file as an output, containing complete directed interactions of all modulated sub-graphs identified. This file could be imported into Cytoscape ^11^ for visualization. Additionally, a file containing details on all sources and sinks for all modulated sub-graphs is reported. Furthermore, for facilitated visualization in Cytoscape, a node attribute file is provided, containing the source weightage and the log-fold changes of all considered genes.

***Operation.*** The only system requirements to run MSF are Java version 8 and JDK 1.8 or above. The few package dependencies are already been added to the release. The runtime of MSF is less than 10 minutes. To run MSF, the user must provide two text files, one containing the DGEA and the second one containing directed interactions in an adjacency matrix format file. Example files and a detailed tutorial to use MSF has been provided on GitHub https://github.com/Modulated-Subgraph-Finder/MSF.

## Results

### Case study

To demonstrate its usefulness, MSF is applied to RNA-seq data set of primary human monocyte derived macrophages (MDMs) infected with Ebola virus (GSE84188) ^13^. Ebola Virus (EBOV) belongs to the Filoviridea family: filamentous, enveloped and single stranded RNA viruses. EBOV causes hemorrhagic fever in humans, inducing the host innate and adaptive immune response to be unable to control virus infection ^14^. Currently, there are no approved antiviral drugs for the treatment of Ebola virus infection ^15, 16^. The initial targets of EBOV are the macrophages and dendritic immune cells ^16, 17^. EBOV inhibits the critical innate immune response of the host, which includes inhibiting the activation of alpha/beta interferon (IFN- *α*/ *β*) ^14, 15, 18^. It has been proposed that IFN- *α*/ *β* should be tested against Ebola for its antiviral activity through clinical trials ^15^. Ebola infection data was selected to test the approach because it has been well recognized for the last several decades, and vast literature is available for the pathogenesis of Ebola, hence facilitating the verification of the results of MSF with the vast literature present on Ebola infection. Especially, the detection of IFN- *α*/ *β* as point of action for the virus could be considered as a basic indicator of the correctness and usefulness of the approach.

EBOV infection sequenced reads count data was downloaded from GEO (GSE84188), it describes the course of infection at three time-points 6, 24 and 48 hour post infection (hpi). Differential gene expression analysis was performed on the count data with edgeR package (version 3.4.2) ^19^ using an upper-quartile normalization. The DEG analysis results generated by edgeR were used as input for MSF. Cell signaling interactions were filtered from Reactome Functional interactions (FIs) Version 2016 ^20^ for only direct interactions, which was used as a second input for MSF.

For the earliest time point at 6 hpi, three large modulated sub-graphs were identified with 42, 139, and 69 genes. The modulated sub-graphs consist predominantly of cytokines and chemokines (CXCL10, CCL8, CXCL9, CXCL11, CXCR4, CCR7, CCL4L1, CCL3L1, CCL4, CCL8, CCL20, CCL3, CCL19, IL6, IL27, IL23). IFNB1 and IFNA1 were both identified as two of the possible sources in the most significantly modulated sub-graph identified with 42 genes. IFNB1 has the highest impact score of 14.5. IFNA1 has an impact score of 8.7, in top 5 highest impact scores in the sub-graph it belongs to. Most of the sources identified by MSF were type I interferon induced genes ( Supplement material). At 24 hpi seven modulated sub-graphs were identified with four main sub-graphs consisting of 61, 222, 130 and 242 genes, others being smaller than 6 genes. Again, IFNB1 and IFNA1 were identified as two sources out of the total sources with 3.9 and 1.6 impact score. IFNB1 was one of the top 5 impact score sources for the corresponding sub-graph. For the last time-point 48 hpi, six modulated sub-graphs were identified. Three of the sub-graphs were less than ten genes and main sub-graphs had 217, 224 and 276 genes. IFNB1 and IFNA1 were identified as sources in the most significantly modulated sub-graph with an impact score of 2.8 and 3.7, but not among the highest ranked sources ( Supplement material).

As stated earlier IFN- *α*/ *β* was reported to be one of the target genes of Ebola infection. We were able to successfully identify IFNA1 and IFNB1 as sources in all three Ebola infection time-points. Although IFNA1 and IFNB1 were already among the most significant genes in the DGEA during the later time points, MSF was also able to detect them as a source in the very early time-point when the genes were not significant based on the individual DGEA alone. Identifying the possible sources will reduce the search space for potential target genes and can help the biologist as the starting point of clinical testing for drugs and vaccines against an infection.

Table 1 compares the results of MSF, namely the number of detected sub-modules and their total genes numbers, to a simple analysis of mapping significantly modulated genes from the DGEA to the network and joining neighbors to modules. The numbers indicate that MSF detects less but larger and easier interpretable submodules, applying its statistical test. Furthermore, the dependency of the results from the *p*-value cutoff choice is demonstrated for the DGEA, which is avoided for MSF altogether. It showcases how applying different cut-offs to the *p*-value of genes from edgeR to the sub-graphs identified by MSF breaks the larger sub-graphs to many smaller unconnected sub-graphs, many of which are single genes.

**Table 1.** Comparison of connected sub-graphs of modulated genes in the global network identified after the analysis with MSF and applying different *p*-value cut-offs from edgeR to genes in MSF identified modulated sub-graphs.

	Total number of genes in networks	Number of connected sub-graphs in network
**6hpi**		
edgeR + MSF	250	3
p-value ≤ 0.1	166	87
p-value ≤ 0.05	152	89
p-value ≤ 0.01	125	76
**24hpi**		
edgeR + MSF	656	7
p-value ≤ 0.1	457	183
p-value ≤ 0.05	418	198
p-value ≤ 0.01	332	216
**48hpi**		
edgeR + MSF	744	6
p-value ≤ 0.1	514	189
p-value ≤ 0.05	468	206
p-value ≤ 0.01	363	241

### Modulated sub-graphs at 6 hpi

**Figure 2.**
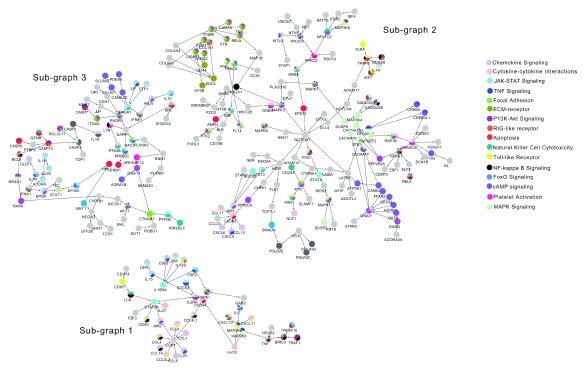
Visualization of the three modulated directed sub-graphs identified by MSF at 6 hpi. The node coloring is associated to KEGG pathways referring to the colors in the legend. The graph edges are from Reactome.

Three main modulated sub-graphs identified by MSF at 6 hpi are shown in Figure 2. The graphs represent the immediate output of the MSF analysis, visualized by StringApp ^10^ in Cytoscape ^11^. Each node represents a gene part of a modulated sub-graph, whereby the associated colors code the functional annotation deduced from KEGG Pathways. The cross-talk between the pathways and also the multiple employment of many genes is evident. The flow of information between the sensors and effectors can be perceived given the directionality of each interaction, indicated by arrows. In more detail, sub-graph 1 (bottom) shows how the activation of Toll-like receptor, Cytokine, Chemokine activating Jak-STAT and MAPK genes, together with TNF leads into apoptosis. The next significant sub-graph (sub-graph 2: top right) reveals how information from the Extra-cellular matrix (ECM) receptor, which are reported to interact with Ebola glycoprotein (GP) ^21^, Chemokines, Cytokines, and Cytosolic DNA sensing are directly or indirectly controlling cell growth, differentiation, proliferation and apoptosis. It suggests that dysregulation of these pathways is responsible for modulation of apoptosis. Eventually, sub-graph 3 (top left) demonstrates how IFNA1 and IFNB1 modulates once more, via only a few intermediate steps, the apoptotic response of the cell. On the other side cAMP signaling genes activates platelet genes.

This display case might advertise with how little effort complex data can be processed and prepared for interpretation by the domain expert, to apprehend the dynamics of the underlying processes and suggest testable hypothesis and potential points of intervention.

### Robustness

**Figure 3.**
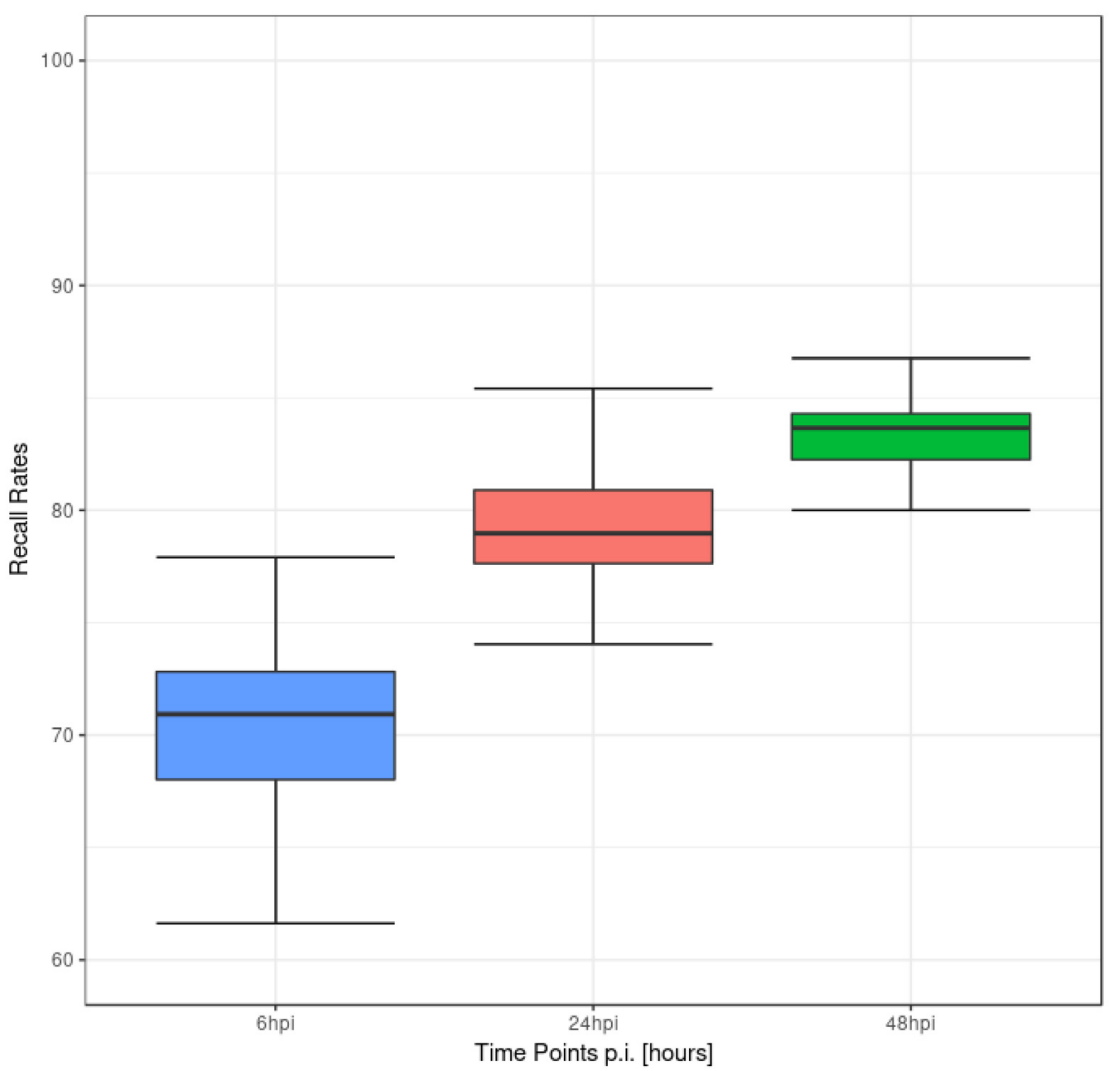
Recall rates for genes in MSF identified sub-graph for the three different time points of EBOV infection data for 100 simulations where Poisson distributed noise was added to the experimentally deduced reads per gene.

A potential concern is how noise in the gene expression measurements affects our analysis. To assess the robustness and stability of our method, we therefore added Poisson distributed noise to the read counts of the three time-points data set, used above. Then DGEA was carried out on the disturbed data with the same parameters as for the native data using edgeR, followed by analysis with MSF. This procedure was carried out 100 times. Every time the genes from the modulated sub-graphs identified from noisy data were compared to the genes of sub-graphs identified from the native data. The robustness of MSF analysis for the time-point 6, 24, and 48 hpi is shown in Figure 3. The procedure how data noise was modeled can be considered as rather stringent as MSF is sensitive to *p*-value changes across the whole range of possible values. The observed median recall rates lay between 71 % (6 hpi) and 84 % (48 hpi).

### Benchmark

The purpose of the comparisons to existing tools is to show the overall capabilities of MSF. MSF was compared to jActiveModules ^8^ since it uses similar approaches to find and score modules. For comparison to classical pathway enrichment analysis Reactome ^5^ and gene set enrichment analysis (GSEA) ^22^ was chosen since both are widely used and the latter does not rely on *p*-value cut-off.

***jActiveModules.*** jActiveModules ^8^ is a plugin in Cytoscape that searches for molecular interaction network to find expression activated sub-networks. The method used to score the expression activated sub-networks is close to the method used in MSF. The difference is in how these sub-networks are identified. MSF starts building the sub-graphs from one gene, incorporating and combining the *p*-value of the next gene, with the check that the combined *p*-value of new sub-graph should be better than the original. On the other hand jActiveModules first transforms all the gene’s *p*-values *p _i_* to z-scores using z *_i_* = Φ ^−1^(1− *p _i_*), where Φ ^−1^ is the inverse normal CDF and tries to find connected sets of genes with unexpectedly high levels of differential expression, in this case high z-scores. The overall score of the sub-network is calculated by combining the z-scores of the genes. Next using their extended simulated annealing method jActiveModules toggles multiple nodes to merge additional components.

**Figure 4.**
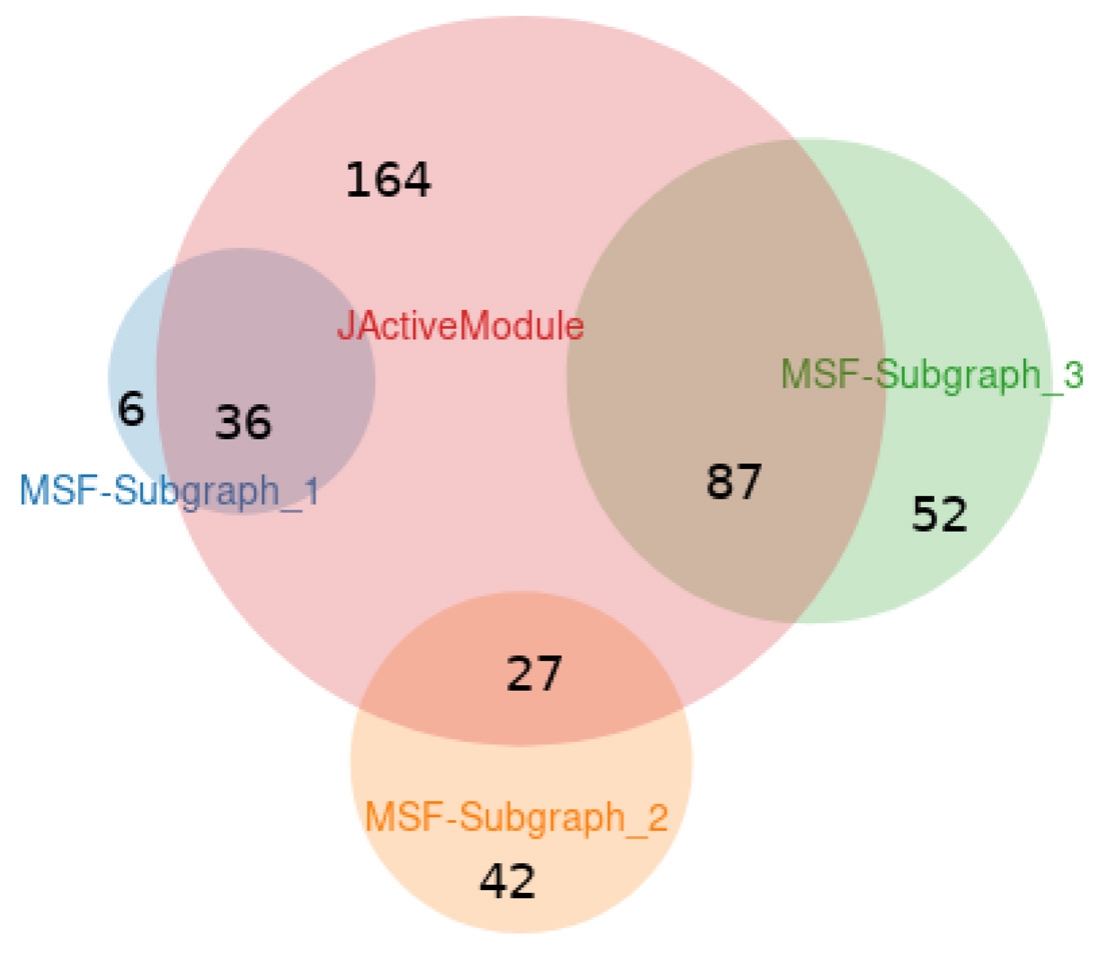
The Venn diagram shows the common genes identified as modulated from MSF identified sub-graphs and jActiveModules identified module.

The first time-point of Ebola infection data was analyzed using jActiveModules (Version 3.2.1) to compare the modulated sub-graphs identified by MSF and jActiveModules. The input files were same for both tools. From the modules identified by jActiveModules, the module with the highest pathScore was selected for comparison with MSF identified modulated sub-graphs. The module consists of a single graph with 314 genes. While MSF identified three directed modulated sub-graphs with 42, 69 and 139 genes. The overlap of the common genes identified between MSF and jActiveModules is shown in Figure 4. The sub-graphs identified by MSF are more fragmented than jActiveModules. Unfortunately there is no golden standard example data that could help benchmark the method. MSF provides directionality, with the identification of possible perturbation sources of the sub-graphs. The predefined pathway labels could also be seen in MSF identified sub-graph with little effort using StringApp ^10^.

**Figure 5.**
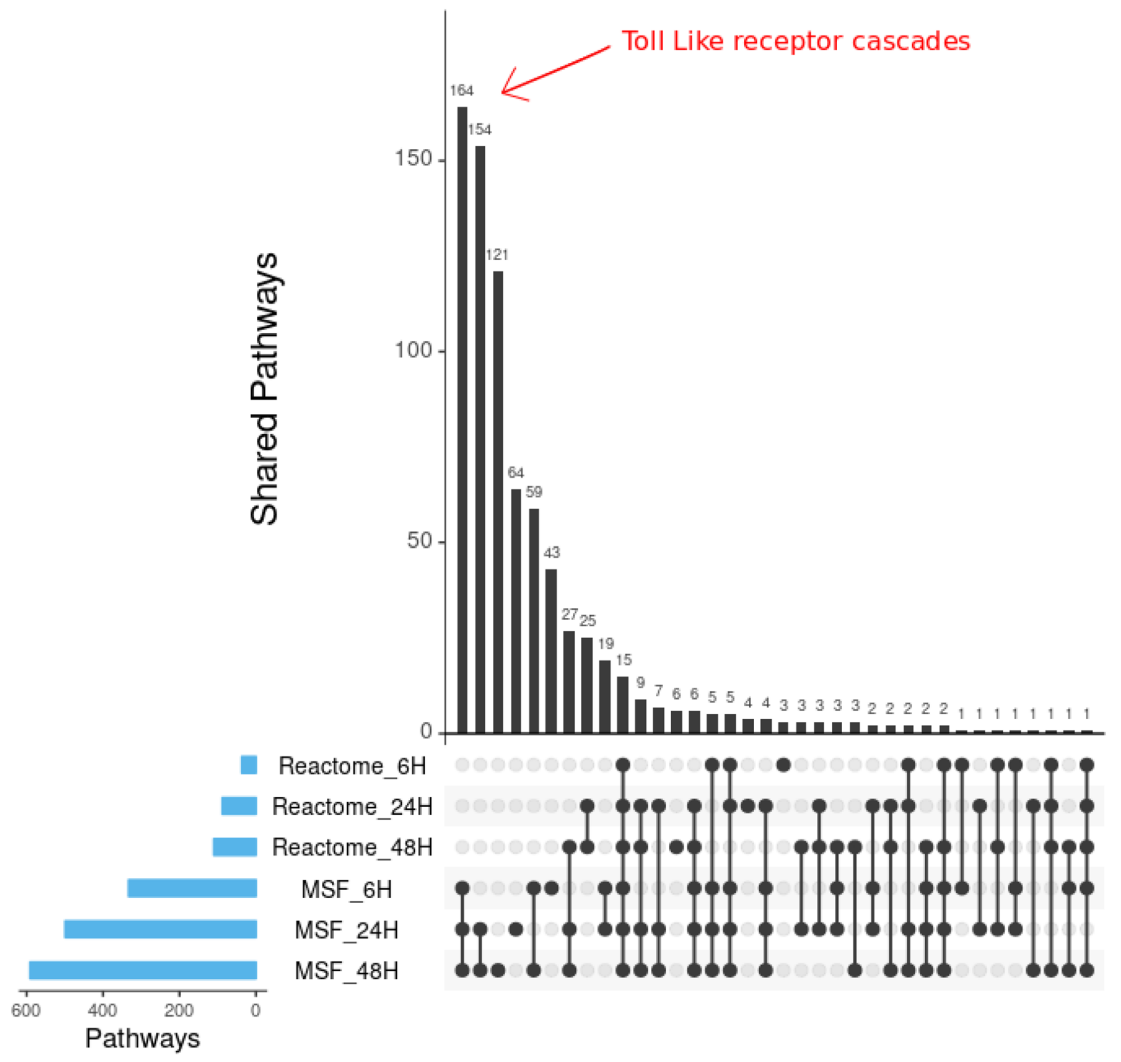
The Upset plot shows the number of shared pathways between MSF identified sub-graph gene list and DEG cut-off list for the three time-points. All 10 different Toll-like receptor cascades are in the set of 164 shared pathways only between MSF at different time-points.

***Reactome pathway analysis.*** Gene enrichment analysis was performed using Reactome analyze data tool ^5^ (version 67) on the different time-points of Ebola infection data. Reactome’s over-representation analysis tool tests whether certain Reactome pathways are enriched for the lists of genes submitted to it. Genes from MSF identified sub-graphs for each time-point were analyzed for gene enrichment using this tool. For comparison the DEG results from edgeR for the three time-points were filtered using the cut-off of adjusted *p*-value < 0.05. These DEG lists were used for gene enrichment analysis. The compression of MSF identified sub-graphs gene lists and the DEG lists analysis is shown in Figure 5.

All enriched pathways with a cut-off of *p*-value <0.05 for MSF and DEG lists for the three time-points were selected. The comparison shows most of the pathways known from literature to be dis-regulated by Ebola infection are enriched in both the enrichment analysis. EBOV glycoprotein (GP) interacts with the Toll-like recpetor signaling pathway, it triggers the activation of cytokines ^13^. Toll-like receptor pathway is expected to be dis-regulated in the early stage of infection, this pathway was not identified as significantly dis-regulated when *p*-value cut off DEG lists were analyzed for enrichment. Nine Toll-like receptor cascades TLR10, TLR2, TLR3, TLR4, TLR5, TLR7/8, TLR9, TLR1:TLR2 and TLR6:TLR2 were identified as dis-regulated from gene enrichment analysis of MSF identified sub-graph genes, not a single one of these cascade was shown to be dis-regulated in pathway enrichment analysis from DEG cut-off lists. Since MSF considers the complete DEG results, even the weak signal at the earliest time-point was detected; for example Toll-like receptor signaling. While MSF is able to catch weak signals, it does not provide information about the functional relationships among genes like Reactome tool.

***Gene set enrichment analysis.*** Gene set enrichment analysis (GSEA) ^22^ is a method to identify classes of genes or proteins that are overrepresented in a large set of genes or proteins. GSEA uses statistical approaches to identify significantly enriched or depleted groups of genes. The complete DEG list from DGEA of the first time-point 6 hpi was analyzed using bioconductor package GSEABase (version 1.44.0). GSEA was able to identify Toll-like receptor, chemokine signaling pathway, cytosolic DNA-sensing pathway, Jak-STAT signaling pathway, RIG-I-like receptor signaling pathway and apoptosis as the highest ranked pathways. Although GSEA identified the important pathways for Ebola infection, it did not show the topology of the genes in the different pathways identified and how they cross-talk. MSF and GSEA uses complete DEG list without any cut-off, that is why pathways important for Ebola infection showed up even with weak signal genes in it.

## Discussion

Classic pathway analysis tools aim to detect in lists of significantly deregulated genes enriched associations with pathway genes categorized by their biological function and their interactions. Depending on the tool, the internal pathway topology is considered or neglected all together. Here presented tool, MSF, employs a different approach, by aiming to detect sub-graphs in whole gene regulatory networks which are significantly deregulated in a concerted manner. To this end, neighboring genes in the user provided network are tested for jointly common regulation. Exploiting that each gene’s abundance, although not independent from its neighbors, is measured on its own, sensitivity can be increased by our applied *p*-value meta-analysis, namely Hartung’s method. This potentially enables to call not just significant modulated genes based on the DGEA to be convincingly called to be part of a deregulated gene group. Furthermore, it allows to identify connected sub-graphs, representing the propagation of gene regulation perturbation in the input network. A better understanding of this propagation, especially the critical spots such as sensors, effectors, and hubs, facilitates the projection of potential intervention points, e.g., for drug development. Since MSF only uses interaction information in gene regulation network, but not the functional grouping of the genes into functional pathways, it is especially adapted to discover so called cross-talk between such pathways.

## Conclusions

MSF is a fast and easy to use tool to find concertedly modulated sub-graphs in a given network. Its implementation in Java enables its use across many operating systems e.g. linux and windows. So far the raw output from edgeR ^19^ and DESeq2 ^23^ are supported.

## Data availability

The Ebola infection RNA-seq data set analyzed during the current study are available in the GEO repository (GSE84188) ^13^. The cell signaling network file used is from Reactome Functional interactions (FIs) Version 2016 ^20^.

## Software availability

Source code is available from GitHub: https://github.com/Modulated-Subgraph-Finder/ MSF

Archived source code at time of publication: https://doi.org/10.5281/zenodo.2632973 ^24^

Software license: MIT license.

## Supplement material

Supplementary material is available form GitHub: https://github.com/Modulated-Subgraph-Finder/MSF
