# Supplementary material for: MSF: Modulated Sub-graph Finder
Source: F1000Res. 2019 Apr 14;7:1346. Originally published 2018 Aug 29. [Version 3] doi: 10.12688/f1000research.16005.3 (PMC6446500; doi:10.12688/f1000research.16005.3)
Supplement: Peer review history

# Peer review: Reviewer response for version 3

**DOI:** 10.5256/f1000research.20710.r47211

**Published:** 2019-04-29

**Authors:** Guanming Wu

**Affiliations:** 1 Department of Medical Informatics and Clilnical Epidemiology (DMICE) , Oregon Health and Science University (OHSU), Portland, OR, USA

Almost all my comments have been addressed satisfactorily.

I have read this submission. I believe that I have an appropriate level of expertise to confirm that it is of an acceptable scientific standard.

# Peer review: Reviewer response for version 3

**DOI:** 10.5256/f1000research.20710.r46070

**Published:** 2019-04-15

**Authors:** Stefanie Widder

**Affiliations:** 1 Medical University of Vienna, Department of Medicine 1, Research Lab of Infection Biology, Waehringer Guertel 18-20, 1090 Vienna, Austria

The authors have addressed all comments sufficiently, I therefore recommend the manuscript for indexing.

I have read this submission. I believe that I have an appropriate level of expertise to confirm that it is of an acceptable scientific standard.

# Peer review: Reviewer response for version 3

**DOI:** 10.5256/f1000research.20710.r47209

**Published:** 2019-04-15

**Authors:** Haibo Liu

**Affiliations:** 1 Department of Animal Science, Iowa State University, Ames, IA, USA

Almost all my questions/comments were addressed to a satisfactory degree. Now the manuscript is in good shape.

I have read this submission. I believe that I have an appropriate level of expertise to confirm that it is of an acceptable scientific standard.

# Peer review: Reviewer response for version 2

**DOI:** 10.5256/f1000research.20442.r46068

**Published:** 2019-04-02

**Authors:** Guanming Wu

**Affiliations:** 1 Department of Medical Informatics and Clilnical Epidemiology (DMICE) , Oregon Health and Science University (OHSU), Portland, OR, USA

This revision has been improved a lot regarding the MSF software and the analysis and discussion of results. This reviewer appreciates the efforts the authors have made to improve the manuscript and the software tool. However, one of the previous major concerns about poor writing has not been addressed carefully. Many typos, grammar errors (especially related to correct use of singular and plural forms of nouns and followed verbs), and other types of errors still exist. There are many examples, here are just some:

  

1. In Abstract, “have proved tobe..”should be “have proved to be…”
2. In Abstract, “changesdue to” should be “changes due to”
3. In the first paragraph of Introduction, “during an infection providing…” should be “during an infection, providing…”
4. “Peerreviewed” should be “Peer-reviewed”
5. “…and combining it to functional pathway annotations” should be “…and combine it …”
6. “regulating each others expression” should be “regulating each other’s expression”
7. “to each a impact score and a measure of its reliability is assigned” should be “to each an impact score and a measure of its reliability are assigned”
8. “git hub” should be “GitHub”
9. In the equation for t(p), lambda in the first term in the denominator should have a subscript i.
10. “default 2 gene” should be “default 2 genes”
11. “the combined p-value of 3 the merged sub-graph”: should 3 be deleted?
12. “containing the source weightage and the log-fold chances of all considered genes.” Should be “containing the source weights and the log-fold changes of all considered genes.”
13. “…use MSF has been provided on git hub” should be “…use MSF have been provided on GitHub”
14. “the detection of IFN-α/β as point of action for the virus, could be”: comma should not be used
15. Table 1: “Number of connected sub-graph” should be “Number of connected sub-graphs”
16. “MSF was compared to jActiveModules8 since they use similar…” should be “MSF was compared to jActiveModules8 since it uses similar…”
17. “jActive-Modules” should be “jActiveModules”.
18. In the “Reactome pathway analysis” section: Please make sure “lists” are used in many places (not list).
19. “The here presented tool, MSF, employs a different approach…”: Delete “The” please

 Other comments: 

1. In the 'Abstract', authors pointed out “These methods … rely on binary separation into differentially expressed gene and unaffected genes based on an arbitrarily set p-value cut-off.”. However, as authors correctly described, the second type of classic pathway analysis approaches (e.g. GSEA) doesn’t do this. This should be changed.
2. T-test is usually applied for data having normal distribution or close to normal distribution. Using t-test for pvalues to calculate source genes’ significance is questionable. 
3. In the “Case Study” section, “The modulated sub-graphs consist predominantly of Cytokines, chemokines (CXCL10, CCL8, CXCL9, CXCL11, CXCR4, CCR7, CCL4L1, CCL3L1, CCL4, CCL8, CCL20, CCL3, CCL19) and Interleukin genes (IL6, IL27, IL23).”: Interleukins are a type of cytokines. Therefore, this sentence should be modified.
4. Human genes should be in upper case. However, genes listed in the Supplement-Material use lower case, for example, https://github.com/Modulated-Subgraph-Finder/MSF/blob/master/Supplement-Material/6H/SourcesAndSinks.text. This should be changed.

I have read this submission. I believe that I have an appropriate level of expertise to confirm that it is of an acceptable scientific standard, however I have significant reservations, as outlined above.

# Response

**Published:** 2019-04-15

**Authors:** Mariam R Farman

**Affiliations:** University of Vienna, Austria

We would like to thank the reviewer once again for the suggestions.

1. All the text modifications and rephrasing has been done.
2. The sentence in the abstract contradicting to the approach of GSEA has been changed.
3. T-test is now performed on the log-transformed p-values of the individual genes, since log-transformation brings the data close to normal distribution.
4. The sentence in the case study regarding interleukin genes has been changed.
5. MSF now outputs the human genes in upper case.

# Peer review: Reviewer response for version 2

**DOI:** 10.5256/f1000research.20442.r46069

**Published:** 2019-04-01

**Authors:** Haibo Liu

**Affiliations:** 1 Department of Animal Science, Iowa State University, Ames, IA, USA

The 2nd version is improved much over the first version in readability and completeness. However, there are still quite a few language issues, unclear statements, and improper expression of concepts. See the commented manuscript in the PDF format for details.

 In addition, the robustness test is not conclusive. Given that RNA-seq data themselves are noisy, I am not sure why it is necessary to add further noise to the RNA-seq data.

 In the 2nd version, the authors compared their MSF tools to two existing tools for network or gene set enrichment analyses of an RNA-seq data from an experimental EBOV infection. But it is not clear how MSF outperform the other two tools.

 The authors claimed their tool is fast, but no runtime is provided.

I have read this submission. I believe that I have an appropriate level of expertise to confirm that it is of an acceptable scientific standard, however I have significant reservations, as outlined above.

# Response

**Published:** 2019-04-15

**Authors:** Mariam R Farman

**Affiliations:** University of Vienna, Austria

Thank you again for your helpful comments about our manuscript.

1. The language issues are thoroughly checked and unclear statements modified.
2. We agree that the RNA-seq data is already noisy, If you look at the robustness of MSF it varies from 71 % (6 hpi) and 84 % (48 hpi), the numbers are not outstanding but we consider them to be reasonable. This shows that adding noise to the already noisy data the robustness is reasonable, if the data is less noisy the chances to get the truly dis-regulated genes increases.
3. We agree that the comparison with jActiveModule was not very conclusive since we did not have a golden standard example data to analyse by both the tools and then compare the results. Although MSF and jActiveModule have the same approach and do find the core genes, MSF additionally identifies the sources of the modulations in the networks which jActiveModule does not provide. The comparison to GSEA showed that although both the tools MSF and GSEA use no p-value cut-off lists and identify the true dis-regulated KEGG pathways, MSF out perform GSEA by providing the actual genes causing the dis-regulation from the lists and shows the cross-talk between the different KEGG pathways in the form of networks which GSEA does not provide.
4. The runtime for MSF has been provided now.

# Peer review: Reviewer response for version 1

**DOI:** 10.5256/f1000research.17481.r40208

**Published:** 2018-11-29

**Authors:** Haibo Liu

**Affiliations:** 1 Department of Animal Science, Iowa State University, Ames, IA, USA

In this manuscript, the authors reported their newly developed tools, MSF, for interpreting gene lists from differential gene expression analysis. Their tool differs from existing pathway analysis tools: (1) it can identify concertedly modulated sub-graphs from user-provided gene networks, thus it can accounting for cross-talk between pathways; (2) it can potentially infer the flow of biological information in response to a perturbation from source to sink. Like the gene set enrichment analysis (GSEA), no arbitrary p-vale threshold is set to dichotomize whole gene lists before applying MSF analysis, instead all genes from DGEA are ordered by p-values from the smallest to the largest. An algorithm similar to the widely used network propagation algorithm is used for subgraph initialization and extension. The authors applied their tools to analyze an RNA-seq dataset from an Ebola virus infection experiment and showed their tool outperform the other tools. They concluded this tool, fast, robust and easy-to-use, is a good supplement to existing pathway-based analysis tools. However, the overall writing is very problematic and there are quite a few issues needing to be fixed. See the details listed as follows. 

1. First of all, the code for the tool is not available via Github. I carefully checked multiple times the Github repository: https://github.com/Modulated-Subgraph-Finder/MSF, however I can't find the ModulatedSubPathFinder.jar file, which is the Java implementation of the proposed tool, MSF. 
2. There are too many grammar issues and writing issues. Just mentioned a few, in the first paragraph of Introduction, "mechanism" in Line 6 should be plural, while "stimuli" in Line 14 should be "stimulus", "maybe" in Line 15 should be "may be". Careful proof-reading is strongly recommended.
3. The authors have a few misconceptions. For example, they treated "effectors" and "sinks" equally. In my opinion, effectors include sources, intermediate genes and sinks, i.e., all genes responding to perturbations. The authors think of the significance of statistical tests in the form of p-values as a confidence level of observing an authentic expression change. This might not be correct. Besides small p-values, the magnitude of fold changes is also important metric of authentic expression change. By the way, the fold change of gene expression is always non-negative. The expression of "sign of fold change" is not meaningful. Only log-transformed fold change is signed. 
4. The flow of information/idea is not fluent in many places. For example, at the end of the first paragraph of Introduction, the authors mentioned the KEGG and Reactome  Pathways. Then at the beginning of the second paragraph, they gave a detailed description of the two pathway databases, which might be unnecessary and disrupted the flow to set up the stage to introduce why their tool is necessary and useful. Some information about how their tool was implemented given in the last paragraph of Introduction should be moved to the Implementation section of Methods. Paragraph 3 under the section of “Case Study”, the DEGA results might better be described immediately after the second sentence of Paragraph 2. Similarly, some information in Conclusion should be move to the section of “Implementation”.
5. The title for the section of "Initial modulated sub-graphs” should be “Initializing modulated sub-graphs “. Under this section, “starting with the most significant one” should be “starting with the next most significant one”. “… not yet in a significantly modulated …”  should be “…not yet in the significantly modulated …”.
6. Under the section of “Extending modulated sub-graphs”, it is not clear how the sub-graphs are extended by adding “MORE THAN ONE” gene at a time.  If doing this way, there are infinite possibilities. The criterion to accept or reject added genes is not clear.
7. Under the section of “Merging modulated sub-graphs”, the authors mentioned that “After detection and extension of the modulated sub-graphs, they are tested if combined subgraphs SCORE better than on their own.” At this point, no merging has been done yet, how are the combined subgraphs tested? What is the SCORE used here? How can a depth-first search traverse from the FIRST sub-graph to the SECOND sub-graph before they are merged (Aren’t the subgraphs not necessarily connected?)?
8. Under the section of “Finding sources & sinks”, “circular loops” should be just “loop”.
9. Paragraph 2 Under “Case Study”, some details about edgeR-based DEGA are missing. How the directed cell signaling interactions ere filtered from the Reaction FIs?  Based on what?
10. The directions of edges in right panel of Figure 2 should be showed, because the MSF can generate directed subgraphs.
11. In the last paragraph of the section of “Modulated sub-graphs at 6 hpi”, “This show cast example…” should be “This show case example…”.
12. The Robustness test seemed to show that the MSF is not robust enough to extra noise. What is the authors’ conclusion and explanation?
13. The authors compared the results from their tool to those from the Reactome pathway analysis tool and demonstrated better performance of their tool. Can they also compare the results from their tool to those from the GSEA tools, which don’t set arbitrary cutoff beforehand? The latter comparison might be more convincing.
14. In Discussion, what are “intermediate bottlenecks”? In Conclusion, the authors claimed their tool is “fast, robust and easy-to-use”. However, they did provide evidence to show their tool is fast. The robustness of their tool is not apparent.
15. Issues with figures: all legend titles are too long. In Figure 1, Texts in the flow chart symbols are not well-written. There are inconsistent case issues; “initial” should be “initializing”; symbol for condition test (“checked all interaction?”) should be diamond, not hexagon. Question marks might be added to condition test for readability. In the legend to Figure 1, what does “without exhaustively testing all connected subgraphs” mean? Does it the output from MSF analysis might not be comprehensive?  Legends to Figure 3 and 4 are poorly written. Is the Toll-like receptor signaling one of the 149 shared pathways in Figure 4. It is not clearly described in the legend.
16. Limitations of their tool:  In their implementation, the fold changes and direction of changes are not taken into account. If a resulting subgraph contains both down-regulated genes and up-regulated genes, how should the users interpret it? The authors didn’t test the sensitivity and specificity of their tool in this manuscript.

I have read this submission. I believe that I have an appropriate level of expertise to confirm that it is of an acceptable scientific standard, however I have significant reservations, as outlined above.

# Response

**Published:** 2019-03-18

**Authors:** Mariam R Farman

**Affiliations:** University of Vienna, Austria

Thank you very much for your suggestions.

1. So far the ModulatedSubPathFinder.jar was only available under the release tag on git hub (https://github.com/MariamFarman/Modulated-SubGraph-Finder/releases). Meanwhile we added the full source code to git hub.
2. The writing of the manuscript has been carefully checked and improved, especially the examples mentioned by the reviewers.
3. We agree with the reviewer that we used the word “effector” not very cautiously. Therefore, we changed it accordingly. The reviewer is again right to consider the magnitude of the fold change as an important metric of the system behaviour. We consciously ignore it since it is not straight forward to include it in our model, and we consider that the p-value at least partly reflect the magnitude of fold change since it expresses the probability that the observed fold change differs from 1.
4. The suggestions are taken into account and the text was modified accordingly. The details about KEGG and Reactome were considered necessary since later the information from both the databases would be used to showcase the results of MSF.
5. Text modified.
6. The use of extending sub-graph here is if the sub-graph could not be extending any more by one gene because the direct neighbouring genes have high p-values. To avoid producing fragmented sub-graphs, we try to, instead of single genes, append branches of up to three genes simultaneously. Thereby the accumulated signal of the whole branch can compensate for single unfavourable genes. Since we limit the procedure to branches of length three, the number of possibilities to be tested is limited. Again, a branch is only accepted if the overall score of the sub-module is better after extension. Details about criterion of rejection and acceptance of extension was added to the text.
7. The wording has been changed for better understanding of the paragraph describing merging of sub-graphs. The score to pass merging is that the combined p-value of the sub-graph after merging two sub-graphs (including connector genes) is smaller than the individual p-values of the two sub-graphs. The depth first search traverse is used to find connectors between the two sub-graphs to merge them.
8. Text modified.
9. Details about edgeR were added to the text. The interaction file downloaded from Reactome is filtered for only direct interactions . The tutorial on how the file was filtered is also provided on MSF git hub page.
10. The directions have been added to the figure and as a output for MSF to import in Cytoscape for easy visualization for the user.
11. Text modified.
12. While developing the algorithm we believed MSF would not only give insights into the network modulation but could also be used to increase robustness of DGEA of single genes by using additional information from their neighbours. Our analysis disproved this assumption, which we wanted to communicate with this robustness analysis. Given the comments by several reviewers we adapted the corresponding paragraph, omitting the comparison to the DGEA but showing only the overall robustness of our tool which is, after applying strong noise, still reasonable with a median recall rate of 71 to 84%.
13. MSF analysis comparison with GSEA analysis added. Although GSEA was able to find pathways known from literature to be dis-regulated during Ebola infection, it could not show the cross-talk between the different pathways like MSF does.
14. By intermediate bottlenecks we actually meant a gene that is actually connecting a number of sources with a number of sinks and thereby a potential point of intervention if one would like to uncouple the input stimuli from the downstream effects. As stated above the reviewer is right about the robustness, the text has been modified accordingly.
15. The figure legends have been rewritten for better understanding. Figure 1 chart symbols edited, symbols shapes modified as suggested. With the phrase “Without exhaustively testing all connected sub-graphs” we intended to say that not all possible connectors to merge the graphs are tested but sub-graphs are connected with the first connector that passes the threshold. In figure 5, Toll-like receptor signaling is actually in the 164 shared pathways between different time-points of MSF.
16. We agree and are aware that magnitude and direction of the fold changes are important. On the magnitude we commented already further above. For the direction, we would like to mention that its interpretation is not straight forward without further information of the type of interaction between the genes, which is not always available. To illustrate, the up-regulation of an inhibitor and the down regulation of an activator can have the same effect on the network.

# Peer review: Reviewer response for version 1

**DOI:** 10.5256/f1000research.17481.r40009

**Published:** 2018-11-12

**Authors:** Stefanie Widder

**Affiliations:** 1 Medical University of Vienna, Department of Medicine 1, Research Lab of Infection Biology, Waehringer Guertel 18-20, 1090 Vienna, Austria

The authors present a novel method for finding groups of genes that concomitantly change their expression profile upon signals and conditional changes. As opposed to standing tools in the field that rely on predefined functional classification, this approach is based on topology of the global interaction network and enables an unbiased identification of larger functional blocks highlighting pathway cross-talk. It furthermore includes a topological method for identifying source and sink pathways that provides a prediction of process causality. The latter is particularly useful for hypothesis generation and add-on experimental validation far beyond the field of biomedicine.

 The paper is structured into the presentation of the algorithm, a biomedical use-case with validating background information and a slim benchmarking against differential expression analysis with different cut-offs.

 While the method clearly fills an existing gap in high-throughput gene expression analysis and is very elegantly reasoned, I would like to raise a number of comments with regards to writing and benchmarking.

Introduction:

1. The main line of argumentation gets lost sometimes, in particular in paragraph 1. The narrative would furthermore benefit from actual examples instead of repeating a general statement of ‘changed conditions’. Sometimes, the same argument is repeated in differently phrased sentences.
2. Paragraph 2: Suppress ‘be subjective to some degree’.  
3. Paragraph 4: Better highlight and delimit the novelty of the presented approach.
4. Overall, shorter sentences benefit the reader.

Methods:

 The context of Hartung’s method is described nicely, yet the actual way how the individual p-values are combined to result in a single measure is omitted. Please include as this information provides instructive benefit to the reader.  

    

Results:

 Case Study:

1. Paragraph 1: Readability would profit from focusing the background information, e.g. 'Ebola infection data was (->were) selected (...)' -  rephrase into a half-sentence.
2. Wording: Until now->currently.

Robustness:

1. The recall-comparison (MSF to differential expression analysis) is weakening the proposed method, because no increase in recall for MSF can be achieved. It would be informative to see also precision stats in comparison. Generally, one would expect more robust statistics on larger subgraphs (MSF vs. DEG groups).
2. Also, I am not entirely convinced by adding noise to real and thus – already - noisy data. A small, artificial mock example with detailed known outcome (+/- noise) might be more suitable and supportive.

Figure 4:

 This figure is very difficult to follow. I suggest to i) enhance the label sizes and texts, with particular emphasis on delimiting MSF and DEG, ii) improve the figure caption text including fundamental information of what is shown.   

 Github material:

 Importantly - provide an example directory that contains complete examples cases (+*all* files) including the presented Ebola case + options and outcome to enable a swift recapitulation of the tool for the new user.

     

I have read this submission. I believe that I have an appropriate level of expertise to confirm that it is of an acceptable scientific standard.

# Response

**Published:** 2019-03-18

**Authors:** Mariam R Farman

**Affiliations:** University of Vienna, Austria

Thank you for your helpful comments about our manuscript.

1. Paragraph 1 has been rephrased, “changed conditions” has been replaced with example of treated verse healthy example.
2. Paragraph 2 modified accordingly.
3. The novelty of MSF is provided in paragraph 5. Firstly, MSF does not use the predefined sets of genes to find the modulated sub-graphs but starts building the sub-graph by using the information from DGEA and interactions from whole cell signaling network. Second MSF considers the signal of the neighbouring genes to find significance of the modulated sub-graph.
4. We revisited the overall text and tried to emphasis more on clear readability.
5. More details about Hartung’s methods are provided in the appropriate section.
6. The case study is rephrased and wording modified.
7. The reviewer is correct about the recall-comparison weakening the proposed method. We have been very strict with testing the robustness of the method by adding extra noise to already noisy data. We expected the method to be more robust than DGEA but unfortunately that was not the case. Since for cut-off based DGEA robustness it does not matter if a few genes p-values go up or down as long as they are below the chosen cut-off. In contrast, for MSF the robustness analysis showed that it makes a difference for the sub-graphs identified. The reviewer is correct again to expect more robustness in the larger sub-graphs which is shown at later time-point 48 hpi that has larger sub-graphs than the other two time-points. If you look at the robustness of MSF alone it varies from 71 % (6 hpi) and 84 % (48 hpi) , we agree that the numbers are not outstanding but we consider them to be reasonable. Since our assumption that a post analysis with MSF can not only gain insights into the pathway modulation but also improve the DGEA for single genes, using their neighbours information did not hold, we removed that aspect to improve readability.
8. We agree that the data is already noisy even before adding noise. Again, we have been very strict to test the robustness of the method,. As mentioned above the larger the sub-graphs the more robust they are, we are not sure that having a small mock example would elaborate more.
9. Figure 5 (previously figure 4) set-up has been changed to one cut-off only i.e. 0.05. Figure label size and texts enhanced. Figure caption modified.
10. Unfortunately we can not provide the Ebola count files since they belong to Olejnik *et al.* **A tutorial is provided to reproduce the results from the Ebola infection data, with details on how to obtain the raw data used from the third party source (GSE84188). MSF output files are also provided in the supplementary material.

# Peer review: Reviewer response for version 1

**DOI:** 10.5256/f1000research.17481.r39844

**Published:** 2018-11-06

**Authors:** Guanming Wu

**Affiliations:** 1 Department of Medical Informatics and Clilnical Epidemiology (DMICE) , Oregon Health and Science University (OHSU), Portland, OR, USA

In this manuscript, Farman et al described an approach to search for network modules based on p-values collected from differential gene expression analysis to address pathway crosstalk, which cannot be addressed in conventional pathway enrichment analysis approaches. During the past decade, many network module-based approaches have been developed to understand the functions of genes collected from differential gene expression analysis and other omics approaches (for a review, see Mitra et al, 2013 ^1^). Though the network approach described here has some innovative ideas (e.g. searching for sources and sinks in subgraphs), however, the authors introduce their approach in the context of pathway analysis, without mentioning these previously published similar approaches, let alone comparing their approach to others. Also, it is worthy of mentioning that the described approach in this manuscript is very similar to jActiveModule (Ideker et al, 2012 ^2^), the first of this kind, widely used for network-based data analysis. 

 The manuscript used the Ebola virus (EBOV) time course gene expression data set to show case MSF, trying to demonstrate the validity and usefulness of the approach. Indeed, the authors found that IFNA1 and IFNB1 are source genes in subgraphs across multiple time points, as reported by literature references. However, the authors have not discussed other genes in the found subgraphs, though the whole lists of them are provided in their GitHub site. IFNA1 and IFNB1 are among other source genes. The authors should develop a statistic approach to evaluate p-values and FDRs for subgraphs and individual source genes, therefore, providing users a way to choose the most significant genes for unknown phenotypes or biological processes. The current way to showcase the usefulness of the approach is not stringent and may not be useful if too many genes are collected in the subgraphs.

 The authors compared MSF results with raw gene lists based on p-value cutoffs (Table 1). However, Table 1 is not a fair comparison. Only the largest subgraphs are listed for edgeR + MSF, while all subgraphs are listed for raw gene lists (e.g. for 6 hpi, 3 for edgeR + MSF, 39 for edgeR (p-value <= 0.1)). For a fair comparison, all subgraphs should be listed for edgeR + MSF too. 

 Section “Comparison to Reactome pathway analysis tool” and Figure 4 compare results produced by Reactome pathway enrichment analysis for different gene lists. The whole section is confusing. First, the section title is misleading: the comparison is for results generated from Reactome pathway analysis tool, not to that tool. Second, I cannot see too much value in this setting using different adjusted p-value cutoffs for gene lists, probably one (e.g 0.05) should be enough, to reduce the clutter in Figure 4. Third, the authors want to point out MSF can enrich Toll-like receptor signaling pathway, but not the raw gene lists. However, such a comparison has not clearly indicated in Figure 4. The set comparisons include too many gene lists. Fourth, the authors should point out what p-value or FDR cutoff value used to choose pathways from the Reactome analysis tool. It is not correct to choose all pathways listed in that tool for comparison. Finally, where “ **Ten**” toll-like receptor cascade pathways come from?

 Searching for sources/targets in individual MSF subgraphs based on the directions in the Rectome FI network and then drawing the schematic diagrams as illustrated in Figure 2 are interesting. It will be better to show directions in the Cytoscape network view (the right-side networks in Figure 2). The schematic diagrams in Figure 2 are interesting, but may dramatically simplify things occurring inside cells. The Reactome FI network provides functional relationships among genes or proteins, which are not necessary gene regulatory relationships. The authors should point this out in the manuscript.

 Finally, the writing of this manuscript is under question. The authors should really read their manuscript much more carefully. There are far too many typos, wrong uses of punctuations, and grammar errors. For examples, “the Filoviridea family; filamentous” should be “the Filoviridea family: filamentous”; “pathogenesis of Ebola. Thereby facilitating” should be “pathogenesis of Ebola, thereby, facilitating”; “among the most significant gene in the DGEA” should be “among the most significant genes in the DGEA”, and many others.

I have read this submission. I believe that I have an appropriate level of expertise to state that I do not consider it to be of an acceptable scientific standard, for reasons outlined above.

## References

[1] MitraKCarvunisARRameshSKIdekerT: Integrative approaches for finding modular structure in biological networks. *Nat Rev Genet*.2013;14(10) : 10.1038/nrg3552 719-32 10.1038/nrg3552 24045689PMC3940161

[2] IdekerTOzierOSchwikowskiBSiegelAF: Discovering regulatory and signalling circuits in molecular interaction networks. *Bioinformatics*.2002;18 Suppl 1:S233-40 1216955210.1093/bioinformatics/18.suppl_1.s233

# Response

**Published:** 2019-03-18

**Authors:** Mariam R Farman

**Affiliations:** University of Vienna, Austria

First of all, we would like to say thank you for your valuable review and your critical comments.

1. Thank you very much for making us aware of the paper by Mitra et al and work by Ideker et al. Frankly, these papers skipped our attention. Meanwhile, the review and the tool JactiveModules has been cited in the paper. Using the same expression data and same interaction file we compared the results from MSF and JactiveModules. Although the methods to find the overall score for the modules is similar, there are differences in the sub-graphs identified. The differences seen in the modules identified by the two methods are because MSF starts building the sub-graphs from one gene, incorporating and combining the p-value of the next gene, with the check that the combined p-value of new sub-graph should be better than the original. On the other hand jActiveModules first transforms all the gene’s p-values to z-scores and tries to find connected sets of genes with unexpectedly high levels of differential expression, in this case high z-scores. And then the overall score of the sub-network is calculated by combining the z-scores of the genes. Then using simulated annealing jActiveModules tries to find the highest scoring modules. Given the observed differences and our focus on the flow of perturbation, namely sinks and sources, we think that our contribution is not redundant to the previous work. To strengthen even further our perspective we also worked on the software itself, which now also scores the sources according to their reliability and the potential impact onto the modified sub-module. Since we believe that this improved the usability of the software critically, we would like to thank the reviewers particularly for point us to this.
2. Again, we thank the reviewer for the valuable input to evaluate the source genes. We acted on this suggestion by amending the software. We have now incorporated an impact score for each source gene, which expresses the percentage of genes in the sub-module which are downstream of the particular source. This should be helpful to prioritize different sources of one sub-module. MSF also performes a t-test for each source gene, testing if the p-values ofthe downstream genes are different from the upstream genes. This would help to see if the source identified indeed marks the border between two different regulation regimes.
3. The table (Table 1) has been modified. It shows number of MSF identified sub-graphs with the number of genes in it. When we apply different cut-offs to p-values of genes in the MSF identified sub-graph, it shows how they break from larger interpretable sub-graphs to smaller, less interpretable sub-graphs also consisting of single genes.
4. Section “Comparison to Reactome pathway analysis tool” has been modified. Figure 5 (previously figure 4) setup has been changed to one cut-off only, i.e. 0.05. Ten Toll-like receptor cascades were seen to be enriched from the genes in MSF identified sub-graphs that did not appear in the cut-off gene list. Since MSF uses no cut-off, the sub-graphs identified had genes from Toll-like receptor cascades even when their signal was weak. Figure 5 caption modified for better understanding. A cut-off of 0.05 was used to choose pathways from Reactome pathway analysis for both MSF and cut-off gene list. The nine Toll-like receptor cascades have been mentioned in the manuscript, tenth being Toll-like receptor itself.
5. Directions have been added to the output of MSF that could be easily imported into Cytoscape. In addition, source impact score and log-fold sign for each individual gene can also be imported into Cytoscape. Functional relationship provided by Reactome FI has been mentioned. The schematic diagram, meant to give our take on the interpretation of the raw MSF output, was removed since we agree on the oversimplification criticism.
6. The writing of the manuscript has been carefully checked and improved.
